# Cognitive Reablement Using Digital Voice Assistants for People Living With Dementia or Mild Cognitive Impairment: A Co‐Design Study

**DOI:** 10.1111/hex.70783

**Published:** 2026-08-02

**Authors:** Tracy Yiannis, Helen Macpherson, David Scott, Michele Callisaya, Caitlyn Gourlay, Ralph Maddison, Eugene Gvozdenko, Paul Jansons

**Affiliations:** ^1^ Institute for Physical Activity and Nutrition (IPAN), School of Exercise and Nutrition Sciences Deakin University Burwood Victoria Australia; ^2^ School of Clinical Sciences at Monash Health Monash University Clayton Victoria Australia; ^3^ National Centre for Healthy Ageing, Peninsula Clinical School, School of Translational Medicine Monash University Frankston Victoria Australia; ^4^ Menzies Institute for Medical Research University of Tasmania Hobart Tasmania Australia; ^5^ Great Australian Pty Ltd Keysborough Victoria Australia

**Keywords:** assistive technology, codesign, dementia, digital health intervention, Digital Voice Assistants, mild cognitive impairment, reablement

## Abstract

**Background:**

Digital health technology has the potential to increase access to home‐based reablement programmes for people living with dementia (PLwD) or mild cognitive impairment (MCI) to support everyday living. Digital Voice Assistants (DVAs) offer a possible solution to overcome usability issues with traditional technologies due to associated cognitive, motor and visual impairments. This paper describes the co‐design of a personalised reablement programme to be delivered via DVA.

**Methods:**

PLwD or MCI (*n *= 9), their care partners (*n* = 9) and health professionals (*n* = 8) participated in the co‐design via online workshops and semi‐structured interviews. Phases 1–7 of the IDEAS (Integrate, Design, Assess and Share) framework guided this iterative process to develop a product for future feasibility testing. Consensus on essential functions and features was facilitated using the MoSCoW prioritisation method. Transcripts were analysed using a modified thematic framework to inform the reablement programme.

**Results:**

Thematic requirements of a cognitive reablement program to be delivered via DVA included (1) Daily Living (self‐care, household and cooking), (2) Activity Participation (leisure activities, home hobbies and ad hoc appointments), (3) Emotional Well‐being (social connection and coping strategies) and (4) technical requirements (activation, adaptable content, adherence, auditory processing, awareness and training).

**Conclusions:**

The IDEAS framework successfully facilitated meaningful engagement from PLwD or MCI, their care partners, and health professionals to integrate user insights and feedback to co‐design a personalised reablement program via DVA for subsequent pilot feasibility testing.

**Patient or Public Contribution:**

PLwD and/or MCI, and their care partners contributed by sharing lived experiences for researchers to gain insights; collaboratively identifying behaviours requiring support; creating ideas for solutions; feeding back on prototypes and prioritising program features and functions. Health professionals fed back improvements on prototypes, identifying potential barriers and solutions to implementation of the program.

## 1 Introduction

Dementia is a degenerative disease involving cognitive and emotional impairment that impact an individual's functioning in everyday life [1]. Mild cognitive impairment (MCI), defined as greater than normal age‐related cognitive decline, can also effect daily functioning [2]. With pharmaceutical treatments limited in scope, the World Health Organisation (WHO) recommends people living with dementia (PLwD) or MCI have access to non‐medical interventions such as rehabilitation programmes to maintain independent living at home [3, 4, 5]. Such rehabilitation programmes encourage involvement from a care partner (CP), such as a family member (i.e. spouse or adult child) or friend, who supports the PLwD or MCI [6].

Reablement is defined as ‘a person‐centred, holistic approach that aims to enhance an individual's physical and/or other functioning, to improve or maintain their independence in meaningful activities of daily living at their place of residence and to reduce their need for long‐term services’ [7]. Unlike rehabilitation, which typically aims to restore lost function, reablement focuses on sustaining independence through goal‐directed support in the home [7]. In the context of dementia, related terms like ‘rehabilitation’ and ‘restorative therapy’ are sometimes used interchangeably, though all involve collaboratively setting meaningful, person‐centred goals with a health professional (HP) and CP, with the PLwD's priorities at the centre [8, 9, 10, 11]. Reablement programmes can therefore encompass a wide range of goals, including leisure activities, social relationships, cognitive functioning, emotional wellbeing and activities for everyday living [8, 12]. Home‐based goal‐orientated reablement programmes demonstrate significant improvements in activity engagement and CP well‐being [10], activities of daily living [13], functional independence and home environment safety [9], and attainment of goal‐related functioning [14]. To facilitate goal‐setting, an assessment guide has been developed that emphasises the PLwD role in selecting the goal, the HP's role in defining the goal within a SMART framework and the CP's role in providing ongoing support [15]. This structured approach helps to navigate potential tensions in goal prioritisation, for example, when the PLwD prioritises social goals, the CP prioritises household activities, and the HP focuses on functional mobility—by anchoring decisions to what is most meaningful to the PLwD. The cognitive demands of this goal‐setting process are thought to explain why reablement programmes have demonstrated greatest effectiveness for people living with early‐ to mid‐stage dementia [9, 16]. Despite this evidence, large‐scale implementation has been hindered by barriers such as training requirements, HPs’ time travelling to patients’ homes and HP capacity [10, 17, 18].

Telehealth has been evaluated as an approach to remotely deliver reablement while overcoming such challenges, demonstrating equally effective self‐ and CP‐rated goal attainment, when compared to in‐person reablement programs [19, 20]. However, telehealth, using traditional digital devices such as laptops, tablets or smartphones, has low usability for older adults experiencing motor and visual impairments [21]. Furthermore, older adults living with cognitive impairments face difficulties in recalling how to access and navigate apps and web‐based programs, making independent use challenging [22, 23]. Digital Voice Assistants (DVAs), such as Amazon's Alexa, that interpret speech and can broadcast audio, images, text and video, offer a promising solution for PLwD or MCI [24, 25]. Emerging evidence for health interventions delivered via DVA has found older adults without cognitive impairment demonstrate significant improvements in memory [26], exercise adherence [27] and social connectedness [28]. Nonetheless, whilst older adults without cognitive impairment have demonstrated successful integration of DVAs into their daily lives, a lack of customisation and user‐friendly interfaces has impeded sustained use [29].

Despite their potential, there is a dearth of research that has investigated the effectiveness of DVA interventions for PLwD or MCI [30]. Previous studies have provided promising results regarding the use of DVAs among PLwD seeking information, with participants preferring the speed and lack of vision requirements of DVAs, and enjoying the conversational voice [31]. Furthermore, a short‐term study found PLwD (*n* = 3) rated the Amazon Alexa DVA as acceptable with a good usability rating despite some communication errors [32]. DVAs provided to older adults, including those living with dementia, by eight local government bodies in the United Kingdom, found they were used for home automation, daily household tasks, appointment management, and social engagement [33]. A 7‐day evaluation of IntraVox, a customised DVA to support PLwD reported successful behaviour change in addition to improving CP wellbeing although usability challenges were identified navigating multiple applications [34]. More recently, technical evaluation studies have found DVAs are feasible and acceptable to deliver cognitive stimulation for PLwD, with findings highlighting the importance of personalisation [35, 36]. Together these findings illustrate the potential of DVAs to deliver reablement programs to support PLwD/MCI.

To facilitate reablement implementation for PLwD, we proposed a program that reduces ongoing HP involvement using DVAs. Together with an industry partner, we have developed a DVA skill (app) called ‘Buddy Link’, which can broadcast personalised evidence‐based health interventions via an Amazon Alexa DVA [27, 37, 38]. Our program retains HPs in the initial critical role of supporting the PLwD, in collaboration with their CP, to establish two or three meaningful goals from a reablement program. This personalised program would then be broadcast via the DVA to support goal attainment over a 12‐week period. Previous studies have shown that incorporating a co‐design process can lead to improvements in content, functionality and design elements, and is recommended for DVA research [39]. More recently, co‐design studies have been extended to include PLwD, demonstrating an increased understanding of their needs and whilst providing opportunities for social connection [40, 41].

To date, there have been no published studies using DVAs for cognitive reablement programs to support PLwD or MCI living at home. This study makes a methodological contribution by establishing that rigorous, iterative co‐design—including active and meaningful participation of PLwD/MCI and their CPs—is both feasible and productive, despite the cognitive and technology usability challenges that have historically excluded this population from digital health development. From an applied perspective, it delivers the first co‐designed cognitive reablement program specifically tailored for delivery via DVAs addressing a gap where no technological solution for reablement currently exists. Conceptually, the study generates new knowledge about the functional, technical and ethical design priorities for voice‐based health interventions in cognitive impairment, including the importance of stage‐specific tailoring and proactive conversational delivery. This paper aims to describe the co‐design process and proposed cognitive reablement program to be delivered via DVA for future pilot feasibility testing.

## 2 Methods

### 2.1 Study Design and Framework

**Figure 1:**
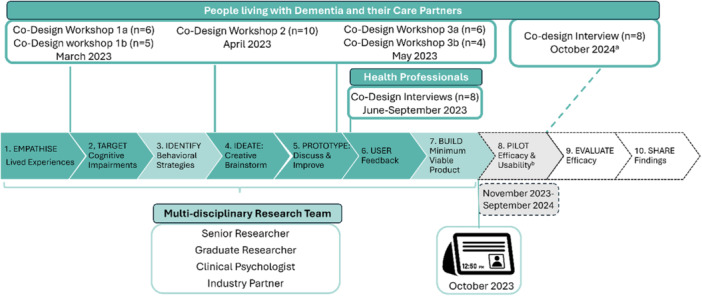
Overview of iterative co‐design workshops and interviews. Steps 1–7 of the IDEAS framework [42] were applied to a minimally viable reablement program to be delivered via Digital Voice Assistants (DVAs). The same participants attended Workshops 1a or 1b, 2, and 3a or 3b. ^a^A second sample of people diagnosed with *early‐stage* dementia or MCI, and their CPs, were consulted post‐feasibility testing to inform a potential large‐scale evaluation (Step 9). ^b^Detailed methodology and results from pilot feasibility testing (Step 8) will be reported elsewhere.

The co‐design of this reablement program delivered via DVA was guided by Steps 1–7 of the IDEAS (Integrate, Design, Assess, and Share) framework [42] as illustrated in Figure 1. The Integrate stage (Steps 1–3) and Design stage (Steps 4–7) involved three co‐design workshops conducted with PLwD or MCI, and their CPs, followed by semi‐structured interviews with HPs. Each stage built on the output of the preceding stage to integrate insights from end users with evidence‐based theory and iterative design with continual user feedback [43]. Following pilot feasibility testing (Step 8), which will be reported separately, the underrepresentation of people living with *early‐stage* dementia became apparent through limited engagement of certain functions and features. Accordingly, a second sample of people living with *early‐stage* dementia and their CPs were interviewed to inform program design for a potential future full‐scale trial (Step 9). Elements of value‐sensitive design were drawn upon in the co‐design process which prioritised the PLwD values of primary importance [44, 45]. To address this methodologically, only PLwD and their CPs were involved in the initial three co‐design workshops to identify reablement‐related goals relevant to them. Empirically, this created an environment for PLwD/MCI and CPs to provide accounts of real‐world reablement experiences and eliminated any potential power imbalances with HPs when identifying conceptually what would inform a program. This initial consultation also allowed identification of the technical needs and capabilities of PLwD that could be addressed and tailored to their values. Once these values had been established within the program, HPs were consulted to ensure their values (e.g., safety) could be included.

This study was approved by the Deakin University Human Research Ethics Committee (DUHREC) Reference: 2022‐043. The Consolidated Criteria for Reporting Qualitative Research (COREQ) checklist [46] can be found in Supporting Information S1: Table 1.

### 2.2 Participants and Recruitment

The target sample size (*n* = 12) for PLwD or MCI, and their CPs, aligned with best practice guidelines for co‐design research for this population [40, 47]. Paid Facebook advertisements, between February and March 2023, targeted older adults residing anywhere in Australia, with a self‐reported diagnosis of dementia or MCI. Given the limited availability of formal, goal‐focused reablement programs for PLwD, it was not feasible to recruit participants with prior experience of structured reablement interventions. As such, the co‐design process centred on understanding participants first‐hand lived experiences, exploring how they naturally adapted their behaviour to remain active in daily life, and the ways in which CPs supported these strategies. Accordingly, initial recruitment did not specify stage of dementia as an inclusion criterion, given the relevance of lived experiences regardless of individuals’ current position along the dementia trajectory. Prospective participants completed an online form via Qualtrics Survey Software (Qualtrics XM) to register for the three online workshops. Inclusion criteria specified adults ≥ 60 years with a diagnosis of dementia or MCI, with a CP also willing to participate and access to Zoom. Following initial screening, a member of the research team (T.Y.) contacted eligible participants to arrange workshop attendance. Given the remote nature of this study, all participants, including PLwD, provided their own informed consent by signing and returning Participant Information and Consent (PLS) forms via email. HPs experienced in working with adults living with dementia or MCI were recruited via snowball sampling using established connections within the research team initially. Recruitment continued until a diverse selection of professionals representing multiple disciplines had been reached ensuring a comprehensive range of clinical perspectives were incorporated.

Following feasibility testing, a second phase of co‐design was undertaken to prioritise the program functions and features. While the initial co‐design included people living with all stages of dementia to capture a broad range of lived experiences, this approach had resulted in an under‐representation of individuals with early‐stage dementia. Given reablement is most effective when applied in the early stage of dementia, a second co‐design phase was conducted using purposeful sampling approaching control group participants from feasibility testing. This co‐design phase focused on prioritising the program content, functions and delivery features to ensure alignment with the needs, capacities and reablement of the target population.

All participants provided online consent to participate in the workshops or 1:1 interview and were compensated with an AUD $100 e‐gift voucher for their time. Two PLwD and their CPs elected to receive a DVA in lieu of the gift voucher. Workshops and interviews were conducted and recorded online via Zoom software (Zoom Video Communications Inc., San Jose, CA, USA).

### 2.3 Workshops

Three sequential co‐design workshops were conducted with PLwD or MCI and their CPs between March and May 2023. Workshops 1 and 3 were offered twice to maximise participation and accommodate participants’ needs (Figure 1). The same graduate researcher (T.Y.) facilitated all workshops and conducted all interviews, a female with qualitative research training and previous interview and facilitation experience, previously unknown to participants. A female psychologist (C.G.) and male senior researcher (P.J.), both with previous qualitative experience, were also present in the workshops to ensure comprehensive data capture and provide support if required. Supporting Information S1: Table 2 outlines the content of each workshop. Throughout the process researchers consulted with a software engineer (E.G.) to address the complex factors of co‐design user requirements with technical feasibility critical for success.

Workshop 1 aimed to understand the lived experiences of cognitive reablement strategies and goals from PLwD and/or their CPs. Using an appreciative enquiry approach [48], participants shared stories where they had successfully used their own reablement strategies, providing researchers with insights on cognitive impairments to target. A reablement goals list [49], selected due to its extensive review of evidence‐based interventions to maintain daily function in PLwD [13], was presented to facilitate discussion. Participants were asked to consider which reablement goals would be most meaningful to them and how a DVA could support them in achieving these goals. Feedback was captured using Zoom whiteboard functionality. Feedback was synthesised from workshops 1a and 1b on the reablement goals and target cognitive impairments that might be addressed via the DVA.

The same participants attended Workshops 2 and 3 to establish continuity and enable iterative building of ideas and concepts. During Workshop 2, video demonstrations of similar DVA functionalities were provided, and participants anonymously rated the usefulness of the potential reablement goals using Zoom voting buttons. De Bono's thinking hats methodology, known for encouraging critical and creative thinking in time‐bound meetings, was used to identify potential benefits and challenges [50]. This technique has been used previously to enhance collaboration between PLwD, CPs and HPs [51].

Workshops 3 involved presentation of ‘quick and dirty’ prototype video demonstrations to gather feedback [52] including detailed digital requirements for the DVA. These low‐fidelity prototypes enabled rapid iteration before significant resources were invested in developing the minimum viable product.

### 2.4 HP Feedback

Ideas and concepts generated from the co‐design workshops were integrated to create prototypes of reablement goals to be delivered via DVAs. HP interviews aimed to gather feedback on each reablement goal to ensure no target behaviours had been overlooked and identify potential barriers to implementation. Interview questions were designed to elicit clinical insights regarding safety, efficacy and practical implementation considerations (Supporting Information S1: Table 2). HP interviews were held separately to avoid any perceived power imbalances that may have arisen in the workshops and potentially suppress idea generation.

### 2.5 People Living with *Early‐Stage* Dementia and Care Partners

Videos of the final reablement goals delivered via DVA were presented during semi‐structured interviews with people living with *early‐stage* dementia and their CPs, who had not previously participated in the workshops. Feedback had to be collated via a series of interviews to accommodate participant availability, demonstrating the person‐centred methodological approach recommended for PLwD. These interviews were conducted as part of the IDEAS framework (Step 6) to gather user feedback and prioritise features for inclusion in a potential large‐scale trial. Supporting Information S1: Table 2 outlines the interview structure with screen sharing software used via Zoom to play video demonstrations of each reablement program goal. Functions were then rated using the MoSCoW method [53] for categorisation on perceived importance and likelihood of using Must‐have (essential functions for the program); Should‐have (important features likely to be used if included); Could‐have (optional features less likely to be used) or Won't have (lowest priority but could be added later). This structured prioritisation approach enabled systematic identification of essential versus optional features, ensuring resources could be efficiently allocated for program development most valued by end users.

### 2.6 Data Analysis

Workshops and interview audio recordings were transcribed verbatim and de‐identified before being inductively analysed by researchers (T.Y. and P.J.) using QSR NVivo 14 (Lumivero, Denver, CO, USA) [54]. Framework analysis was used to compare and contrast transcript data from workshops and health professional interviews across the different stages [55]. Five stages of coding were completed: (i) data familiarisation (reviewing interview and workshop transcripts); (ii) both researchers (T.Y. and P.J.) agreed on an initial thematic framework for coding; (iii) indexing—one team member (T.Y.) systematically coded the data to all interview and workshop transcripts; (iv) charting—both researchers (T.Y. and P.J.) reviewed and refined the themes and sub‐themes from the thematic framework; (v) mapping and interpretation—themes were mapped to the International Classification of Functioning, Disability and Health (ICF; WHO 2001) [56] to evaluate the conceptual coherence of the program components.

## 3 Results

Twenty‐six people participated in this co‐design study (9 PLwD or MCI, 9 CPs and 8 HPs) (Table 1). Most PLwD or MCI were female (78%), median age 71 years (range 66–83), and all had their spouse as their CP. Participants were from across five Australian states, representing experiences from varied healthcare support systems. The mean (SD) duration of the workshops was 72 (±17) min. While not an inclusion criterion, three PLwD and CPs owned a DVA and a further two elected to receive one in lieu of a gift voucher, reflecting a diversity of DVA user experience. The mean (SD) duration of interviews with participants with early‐stage dementia was 55 (± 9) min. Healthcare professionals from a diverse range of allied health and clinical fields were interviewed, encompassing both direct care providers and specialists in cognitive reablement. The mean duration of Health Professional interviews was 67 (± 7) min.

**Table 1:** Participant demographics.

	Workshops (*N* = 10)	Interviews (*N *= 8)	Total (*N* = 18)
People living with dementia or MCI	*n* = 5	*n* = *4*	*n* = *9*
Age, median (range)	71 (66–77)	74 (69–83)	71 (66–83)
Gender, female (*n*, %)	3 (60)	4 (100)	7 (78)
Cognitive Impairment type (*n*, %)			
Mild cognitive impairment	2 (40)	2 (50)	4 (44)
Alzheimer's disease	2 (40)	2 (50)	4 (44)
Frontotemporal dementia	1 (20)	0	1 (11)
Age at diagnosis, median (range)	65 (58–70)	70 (69–82)	69 (58–82)
State/Territory (*n*, %)			
New South Wales	1 (20)	0 (0)	1 (11)
Queensland	2 (40)	1 (25)	3 (33)
South Australia	1 (20)	0 (0)	1 (11)
Victoria	1 (20)	2 (50)	3 (33)
Western Australia	0 (0)	1 (25)	1 (11)
Care partners	*n* = 5	*n* = 4	*n* = 9
Age, median (range)	71 (66‐77)	73 (72‐86)	72 (66‐86)
Gender, female (*n*, %)	2 (40)	0 (0)	2 (29)
Health professionals	(*n* = 8)		
Discipline			
Occupational Therapist	1		
Dementia Nurse	2		
Psychiatrist (of old age)	2		
Psychologist (Clinical/Neuro)	2		
Geriatrician	1		
Gender, female (*n*, %)	6 (75)		
Time in current role, median (IQR)	11.5 (5.4)		
Time as registered clinician, median (IQR)	23.3 (15.3)		

### 3.1 Themes

Three overarching reablement themes were identified from the workshops with PLwD and their CPs, and health professional interviews: Daily Living; Activity Participation; Emotional Wellbeing. These themes and sub‐themes, along with supporting quotes, are summarised in Table 2. Themes were discussed with similar levels of relevance and emerged consistently from PLwD or MCI, CPs, and HPs. Supporting quotes mapped to the final program modules are included in Supporting Information S1: Table 3. A fourth theme, describing DVA Technology Customisation requirements, along with related sub‐themes is outlined in Table 3.

**Table 2:** Consolidated findings from workshops with PLwD and their CPs, and HP interviews across the IDEAS framework stages.

Theme		Empathy	Define	Ideas	Prototype feedback	Minimum viable product: co‐designed module
*Activity participation*: Community activities Ad‐hoc appointments	Supporting quotes	‘We like to have at least one outing a day where we go out and do something, so it's just good to connect with nature and the people around us, our community.’ (PLwD01)	‘it's kind of a more naturalistic form of practising memory, of keeping engaged with the community and what's going on and being able to have, you know, conversations with people and being aware of what's happening’ (HP06)	‘Well for me just a verbal reminder of say dental appointment or doctor's appointment because they're the ones even if you put them in your calendar you can forget.’ (PLwD01) ‘… reminders when you get up in the morning, you know, what you're planning for the day.’ (PLwD01)	‘…those sorts of things [checklists] I think are really important just to motivate them and remind them of the activity’ (HP03) ‘Yeah, that's great. Especially the reminder on what to take, that would be super helpful.’ (HP06) ‘I think the appointment stuff is going to be important. It's a source of embarrassment; it's a source of frustration for carers.’ (HP02)	Activities and Appointment Assistant Customised *reminders* and *checklists* for regular weekly leisure activities. Personalised set‐up and reminders on when and how to use the integrated DVA functionality: – Calendar Appointments – Ad‐hoc Checklists
	Findings	PLwD or MCI want to continue an active lifestyle outside the home.	Reminders and checklists can provide cognitive compensation for executive function and recent memory impairments. Community activities provide valuable social cognitive stimulation.	Audio verbal activity reminders can support memory recall beyond ‘appointment alarms’. Alexa calendar routines could facilitate daily executive functioning.	Use of visual images on checklists provide critical memory prompts required by some PLwD. Set‐up support requested for digital calendar users.	
*Activity participation*: Home Hobbies	Supporting quotes	‘I do have a little garden so that's a big interest of mine… I really enjoy looking after the garden, that's something I do every day, just even if it's looking at the plants or picking a few flowers for the house.’ (PLwD01) ‘I often come across families of people with dementia that want to say continue to knit but they can't remember how to do some of the stitches or continue to crochet but they can't remember how to do it…’ (HP03)	‘… or [they] forget [how] to put on their favourite show, like an animal show, or a travel show or a cooking show, things like that.’ (HP03) ‘I think those are great interventions, … listening to the news, podcast, audio books. And maybe even like interesting documentaries, like David Attenborough documentaries, because those are all things that are stimulating, they are engaging, and they require some level of cognitive engagement.’ (HP06)	‘… like if they've enjoyed travelling and they've gone to a particular country and you have a short clip on, you know, that place. And if they like gardening, maybe they can link that into Gardening Australia. And those types of things are inherently pleasurable to watch… (HP06)	‘…a lot of them have negative self‐talk and they often feel quite poorly about their cognitive abilities and their memories, and often we're really trying to provide a bit of reassurance and trying to see what we can do to minimise that negative self‐talk. That might be a bit at odds with this idea of sort of testing their memory. (HP04) ‘… it's amazing how many people won't be able to remember this although they're not that severe in their cognitive impairment. People often have what we call a catastrophic reaction where they become quite overwhelmed.’ (HP01)	Active Minds Personalised links to weekly YouTube Videos related to Home Hobbies Personalised set‐up and user guide for: News briefings Podcasts Audiobooks
	Related findings	Implicit/procedural memory impairments can lead to make it difficult to maintain home‐based hobbies.	Often task initiation is the barrier for hobbies (e.g., gardening/knitting) that can be adapted. Audiobooks can compensate for complex attention impairment.	Short YouTube videos related to hobbies of interest included. User Guide for Audible (audiobook) function included.	Brain Games excluded from remote DVA delivered reablement program.	
*Daily activities*: Self‐care	Supporting quotes	‘If I forget one day, then I could have a seizure, so I really find it valuable to have a reminder’ (PLwD01) ‘We just take all our medications at breakfast and [name] has to have a tablet as she gets out of bed so I remind her about that every morning.’ (CP03)	‘I used to take four tablets in the morning and then one in the afternoon… So you've got to make sure that you actually detail the actual quantities and the actual specific tablet in the reminder’ (PLwD02) ‘With myself it's important I take my medication every day.’ (PLwD01)	‘… actually eating is like an appointment for some people. Because the stimulus to eat is often not that strong.’ (HP02) ‘…a reminder for drinking water, fluids is really paramount. I have a lot of clients who end up, and one today, who's ended up in the emergency department because they're only drinking 500 mils of fluid maximum a day.’ (HP05)	‘…our community nursing service really can't go in more than twice a day. So, if clients’ needs medication three or four times a day it's just not doable because there's not the resources to stretch that far…… So even if they needed say administration of their insulin in the morning it might still give them the independence in the evening time with the Alexa.’ (HP03) ‘ …. putting a reminder on the tap is fine, but having a regular reminder to drink water would be amazing and I think really beneficial, especially early in their ‐ with MCI or early dementia because hopefully it then becomes a pattern and it becomes a routine and that they actually continue with that as they go along their journey. So, yeah, I think that's a really great tool to have’ (HP05)	Routine Reminders Customized self‐care reminders/instructions:
	Related findings	Many PLwD or MCI take medication for co‐existing conditions. CPs often act as medication reminder.	Medication monitoring beneficial for PLwD or MCI to compensate for short‐term memory impairments. Images with detailed prompts required in addition to generic ‘medication’ alarm reminder.	Medication reminders to include optional personal image (e.g. webster pack). Routine nutrition reminders to be offered to compensate for loss of hunger cues. Hydration reminders added to prevent dehydration.	Encourage medication or webster pack to be kept next to DVA. Establish regular hydration routine early on to develop unconscious behaviour.	Medication Hydration Nutrition
*Daily activities*: Housework	Supporting quotes	‘Can I just say my biggest problem perhaps is using the remotes to record things on the television, at the moment <my partner> does it all, but I'm terrified that if he's not able to do it I wouldn't be able to do it.’ (PlwD01)	‘Well just the procedures or the steps that it takes to, what I need to do, because there's two remotes and knowing which one you use first and which one you turn on first…’ (PlwD01) ‘… the sequence to get my wife used to putting on the lead, the harness and all that sort of stuff. We used that [Alexa] a fair bit in the early stages because she wasn't familiar with that.’ (CP05)	‘I have one gentleman who always puts the saucepans in the wrong place, always, it drives his wife to distraction…’ (HP05) ‘…. routine laundry tasks, they often seem to remain independent, and often the change, I've found, can be more to do with motivation…’ (HP05) ‘Your prompts can come with the motivation I'm thinking. So today is Tuesday, if you wash the sheets today your home care provider, can hang the sheets out, so they'll have the motivation to do it, well to please someone else but to achieve a goal. I think that's a good motivation for a lot of people.’ (HP05)	‘…for a person who's living with dementia, it's not about doing the laundry, is it? It's so much more. I just love it. Putting the clothes out versus in the dryer and the thought process. I actually think this particular scenario is a very great example of where a person would actually be mentally challenged and this would help their cognitive reserve because although Alexa is sort of doing it for them, they still have to actively work with the task.’ (HP01) ‘You have to allow people to have autonomy and quality of life and with that, there will be risk and there is dignity in that risk. As long as the person living with dementia and their caregivers are aware and they give you consent, it's acceptable, even if it leads to an adverse outcome if that was what the person wished for themselves.’ (HP01)	Household Manager Customised step‐by‐step instructions and/or user videos. Kitchen item locator.
	Findings	Remembering how to use complex or new household appliances can be challenging for individuals experiencing learning and memory impairments.	Step by step instructions could compensate for cognitive impairments.	Kitchen item locator could support memory recall. Customised reminders could provide household task initiation and motivation.	Dignity of risk should be considered for the PLwD to continue with autonomy of tasks.	
*Daily activities*: Cooking	Supporting quotes	‘it's a barrier sometimes for social engagement to not take a plate or have something when people come.’ (HP02) … shopping list because we tend to forget things, you know, if we don't write them down or talk to each other about what we need’ (PLwD01)	‘Is there – is there any evidence, qualitatively, about what people get frustrated with when they give up baking? Certainly, in my clinical experience it's usually a missed ingredient or a single missed step.’ (HP02) ‘We tend to stick to much the same menu every day, so we don't forget [laughs] what we like and what we want.’ (PLwD01)	‘I wouldn't go with the apple pie with 15 steps. I think there are some really simple things like sausage rolls and, you know, a chocolate cake which, you know, our kids were making – I would look at, you know, honestly, I'd look at kids’ recipes at primary schools; stuff that kids can do. Because they're usually one container and, you know, six ingredients and you mix them all together and then put it in the oven…’ (HP02)	‘I think the shopping list would be great for my husband because he very often will get to the shop and forget his list and then he'll ring me and say “what was on my list” and I've got to try and find it.’ (CP04) ‘I can see that application being really, really useful in Commonwealth home support packages for people with home care workers, because one of the activities that I ask them to do, for those that are particularly wanting to do recipes, is to come in, to take them shopping, to do their shopping to buy the ingredients, and then to come home and actually help them cook the meal, rather than just going straight to Meals on Wheels or Light ‘n’ Easy.’ (HP05)	Kitchen Assistant
	Findings	Bringing ‘a plate’ [of food] can help maintain confidence at social events with family and friends.	Customised recipes could facilitate autobiographical memory.	Recipes should use minimal ingredients with simple step‐by step instructions to support deficits in complex attention.	User guide for Alexa's integrated Shopping List function to be included in set‐up.	Customised step‐by‐step recipes Alexa User Guide: Shopping List
*Emotional wellbeing*: Family and Friends	Supporting quotes	‘you do notice with people with dementia families will often make comments that their loved one doesn't ring anymore and unless they ring them they don't really ring them and they often don't ring their friends.’ (HP03) ‘… I forget everybody's birthday’. (PLwD01)	‘ I actually have a picture of my family tree next to me on the desk here’ (PLwD01) ‘They might just be ringing one or two family members but obviously as time goes on that cuts out and if they're living on their own and they're socially isolated…’ (HP03)	‘“This is your story” but maybe having another option for a day that's not such a good day of, I don't know … some photos’ (HP01) ‘I think this is great. Because they are more likely to pick up the phone then have that chat with that person’ (HP07) ‘The reminders, the pictures because I think the pictures are obviously important you know names go pretty quickly. Faces are, you know, in terms of memory, obviously stick for longer.’ (HPO8)	‘It sounds – it sounds excellent. Actually, [name] for her 70th birthday my youngest daughter put a book together and gathered all the – from all her siblings, nieces, nephews, the lot. Well, I've got it here now so I could almost put that onto it’ (CP03)	Family and Friends Facilitator
	Findings	Impairments in executive dysfunction can result in PLwD not initiating social contact.	Regular reminders to phone family and/or friends could facilitate social contact that provides protective cognitive stimulation.	Phone Call Reminders to include a ‘conversation prompt’ and photo to compensate for memory loss. User guide to include set‐up instructions for Alexa's integrated photofunction for visual autobiographical cognitive stimulation.	Life Story videos could be an alternative to ‘hard copy’ albums/books to support autobiographical memory recall and provide reminiscent therapeutic benefits.	Reminders to Call & User Guide for Alexa's integrated function Photo User Guide Life Story – weekly reminders to write with final story broadcast.
*Emotional wellbeing*: Coping Strategies	Supporting quotes	‘Well, we've used it a fair bit of music and my wife likes Janis Joplin so I usually say “Alexa, can you play Janis Joplin” and then I go out in the backyard or in the garden and she relaxes.’ (CP05) ‘…we always have music in the background, so that's something we love, and I love the way Alexa can pick a tune that you might love, and it's a great way to start the day with the music.’ (PLwD01)	‘Memory loss is more often than not associated with poor concentration or attempts to multitask or having poor attention or emotionally, being, not in an optimal state. So those situations lead to more profound memory loss than actual brain degeneration due to dementia’ (HP01)	‘In the joke function do you have the option of saying, ‘Hey, remind me about that joke, or remind me – can I – can I remember that joke because I'm going to tell it to my grandkids…, you know, something like that.’ (HP02)	‘…I think if my husband was home alone, and I had preset this for him, I think he wouldn't feel quite – …. sometimes he feels really lost if I'm not here… and if there was something that could help keep him centred and calm, I think that would be very helpful’ (CP04)	Mood Monitor Initial mood reflection question followed by wellbeing intervention options:
	Findings	PlwD or MCI often experience symptoms of anxiety associated with their diagnosis.	A restorative personalised intervention at a prescribed time of day could help manage symptoms. Practicing techniques such as breathing or mindfulness could help regulate emotion to support concentration and memory.	Add function to repeat joke to memorise recall for the cognitive and social benefits as well as therapeutic.	Set‐up to include personalised music, in addition to therapeutic music options, with reminders on when and how to use Alexa's integrated music function.	– Mindfulness exercises – Nature scenes – Jokes – Music

**Table 3:** Technical features and format for DVA deliverl's.

DVA Program Features and Format	Supporting Quotes
*Activation*	*‘What activates Alexa? I was just wondering whether you could start it, almost like, like an alarm.’ (CP05)*
Option for automatic skill broadcasts as an audible conversational prompt (in addition to user initiation);	
*Adherence*	*‘…even if it was a weekly monitoring, just to get some sense – particularly if you've got medications involved, you know, that sort of stuff would be reassuring for family, I think…. it would be reassuring for me as the – as a doctor involved…’ (HP02)*
Option for self‐monitoring and/or emails sent to CP;	
*Auditory and Processing*	‘…a lot of this patient group have quite significant hearing impairments and might often forget to wear their hearing aids unfortunately. How loud can it go?’ (HP04) ‘I think you're thinking mostly amnesic MCI, and the vascular MCIs have very slow processing speed…. You'll need to have some smarts in there to just allow the consumer to say, “Yes, yes, yes, and just get on with it because I don't want to hang around for your stupid questions anymore”’ (HP02)
Volume control Voice speed Instructions to connect hearing aids Omit back‐ground music from instructional videos	
*Awareness, Set‐up and Training*	
Location: Ensure DVA is in a frequently used area (e.g., kitchen) ‘Cheat‐sheet’ of voice commands Set‐up support for Alexa smartphone app Wi‐fi extenders for larger houses Instructional training videos for integrated Alexa functionality	‘And I wondered if it might be a good idea to have a poster of that with, you know, the headings that you had… something that the patients could put near the device. They might not be able to remember it. So, if there is a prompt….’ (HP07) ‘… the how to videos once a week. You learn something [an Alexa‐function] new each week’ (PLwD02) ‘I'd like the how‐to videos on Alexa; I think that would be really helpful.’ (PLwD01)
*Adapt content to user preferences*	‘Everyone responds better to positive feedback and affirmations and acknowledgement of what they can do, and if this helps them…. they won't feel that they're relying on the technology, they will feel that the technology is supporting them. So, just at the end of each little thing, “well done, way to go, awesome!”’ (HP05) ‘…home video maybe combined with a narrated slideshow. Animated doesn't work…. the people with dementia can't relate to avatars, little cartoon figures doing things. But your videos, where you've actually got a person, they're excellent because they're relatable’. (PLwD01)
Personalised motivational message (option) Images: User's own photos/videos or stock icons/photos (***not*** animations/cartoons Video format: Narrated PowerPoint slideshow movies were considered acceptable.	

#### 3.1.1 Daily Living

Daily living activities emerged as a predominant domain to be included in the reablement program reflecting their importance in maintaining functional independence for people with dementia/MCI. This theme primarily focused on supporting cognitive impairments in short term and procedural memory that hindered participants’ capacity to manage self‐care and household responsibilities. Three key sub‐themes were identified: self‐care (e.g., medication, nutrition), household tasks and cooking support.

##### 3.1.1.1 Self‐Care

Many participants lived with co‐morbidities requiring essential medication, but short‐term memory lapses affected adherence contributing to co‐dependency on CPs. Participants requested comprehensive self‐care prompts for medication support, emphasising multimodal cognitive support, specifically the option for visual images in addition to auditory instructions:

… the blue tablet I have to take morning and night, and the brown tablet just at night.

(PLwD02)

HPs emphasised poor hydration was a key safety concern, contributing to increased incidences of urinary tract infections and more severe dehydration complications. Self‐care was also expanded to include nutrition, highlighting the importance of addressing decreased appetite, which transforms eating from a sensory‐driven behaviour to routine activity:

…'How about muesli and a nice yogurt?’… and those suggestions would be predetermined by them as well.

(HP08)

##### 3.1.1.2 Household Tasks

Evolving technological changes in household appliances, from TV remotes to washing machines, as well as non‐technological changes, presented a challenge for those participants who struggled to learn procedural tasks. Participants requested housework activities be included with options of customised ‘how‐to’ prompts for household appliances which could use individual's own photos or smartphone videos. This would enable (re‐)learning of procedural memory tasks, creating a personalised cognitive prosthetic for everyday activities:

…we got new taps, we used to have hot and cold, now it's the mixer tap, and [name] still can't work out which way hot is, which way cold is…and that's really thrown her.

(CP03)

##### 3.1.1.3 Cooking Support

Participants advocated for support with cooking tasks including step‐by‐step recipes to accommodate impairments in complex attention and procedural memory. Personal recipes were considered important, given the reminiscent nature of cooking to support autobiographical recall of familiar activities.

I wouldn't go with the apple pie with 15 steps. I think there are some really simple things…. they're usually one container and, you know, six ingredients and you mix them all together and then put it in the oven…

(HP02)

Learning to use Alexa's integrated shopping list was seen as an invaluable cognitive assistive technology also helping mitigate impact for CPs:

[Name] very often will get to the shop and forget his list and then he'll ring me and say, ‘what was on my list?’ and I've got to try and find it.

(CP04)

#### 3.1.2 Activity Participation

##### 3.1.2.1 Community Activities

Maintaining a lifestyle that included social, mental and physical activities was consistently identified as a priority; however, impairments in executive functioning meant participants required support to independently maintain community participation. Despite the use of memory aids such as lists and calendars, participants remained dependent on CPs to prompt and support their effective use. The importance of these cognitive aids was emphasised to maintain confidence when leaving home, especially for regular scheduled leisure activities.

We have a little checklist every day before we go out of the house, so I don't forget the bus ticket or the mobile phone or the keys

(PLwD01)

##### 3.1.2.2 Home Hobbies

Use of the DVA for cognitively stimulating activities was extensively discussed including learning how to use Alexa's integrated audiobooks and news functions. This was particularly important for those whose complex attention or visual impairments meant enjoying books had become challenging.

…she's always been a strong reader, but I know she can't stay with it, you know, after a paragraph or so…

(CP03)

Podcasts and short videos related to personal hobbies were seen as both instructional tools to support procedural memory deficits (e.g., knitting), as well as motivational prompts to address the executive function impairment:

[name] used to sew… do a lot of sewing. We pulled out the sewing machine 12 months ago, but those skills are gone.

(CP03)

##### 3.1.2.3 Ad‐Hoc Appointments

Participants described difficulties remembering details relating to both essential medical appointments and non‐routine social engagements with family and friends, leading to increased dependence on CPs to support organisation and attendance. In addition to setting up customised features for regular leisure activities, participants requested a user guide on how to use Alexa's integrated calendar, checklist and auditory reminder functions, reflecting a desire for sustained independence through technology adoption.

Although I put it into his calendar on his iPad, sometimes he doesn't look at it.

(CP04)

#### 3.1.3 Emotional Wellbeing

##### 3.1.3.1 Family and Friends

Participants endorsed the idea of a reablement goal to facilitate relationships with family and friends, recognising the critical role of social connectivity in preventing isolation. Learning how to use Alexa's integrated voice‐activated audio and video call feature was included, with customised audio and photo reminders to contact family and friends. Two additional functions were requested as part of this goal, to support preservation of autobiographical memory for maintaining self‐identity and interpersonal relationships with others. The first was using the DVA for reminders to write their Life Story, which would then be broadcast as a narrated video with photos. The second was learning how to use Alexa's integrated photo display function with instructions for adding descriptive text.

I've got a ‘Book about Me’ on [name], you know, right from when she was born and everything else; the highlights all the way through, trips overseas, and all that

(CP05)

##### 3.1.3.2 Coping Strategies

The inclusion of emotional regulating strategies to support individuals experiencing anxiety and depression associated with a cognitive impairment diagnosis was viewed as a valuable addition. This included the use of music to promote relaxation and uplift mood across all stages of dementia, as well as help manage agitation in later stages. Participants liked the idea of automated or voice command music broadcast using the DVA for either customised and/or therapeutic playlists.

She enjoys that type of music. So, it's been very relaxing for her, and she sits there and listens to it

(CP05)

Participants expressed interest in the inclusion of breathing and mindfulness exercises for people living with early to mid‐stage dementia to help manage anxiety related to the impact of cognitive impairments on their daily functioning, such as prior to leaving the house, or at bedtime. HPs were supportive of videos for mindfulness exercises, recognising the growing evidence base for non‐pharmacological approaches for emotional wellbeing in dementia.

I love mindfulness. I think everyone should practise mindfulness.

(HP01)

#### 3.1.4 Technology Customisation

Technology requirements of the DVA program were explored with five key dimensions of customisation emerging as important for consideration to optimise acceptability and usability (Table 3). First, *activation*, that is, voice‐initiation or automated broadcast of an audio conversational reminder rather than unspecified alarm, was highlighted as being a flexible requirement, dependent on an individual's stage and type of cognitive impairment.

Say it's seven o'clock… Alexa automatically says, ‘Hi [name] it's time to take your tablets… Have you thought of it?’

(CP05)

Second, *adherence* and monitoring of daily activities (e.g., medication) were proposed as unique feature to optimise the interactive functionality of the DVA. Alerts for busy CPs were seen as a key benefit rather than a system that required a CP to remember to access and review the data:

Would it send off a message to the carer rather than it being reliant on the carer to check?

(HP04)

*Auditory* processing and hearing impairments were considered critical needs to be accommodated, including adjusting both the DVA device volume and speaking rate to align with individual impairments.

…one of the first cognitive impairments that someone gets is delayed responses, so it takes them a bit longer to do these things…

(HP05)

*Awareness* and training for pre‐installed cognitive compensatory and entertainment features on the DVA device were requested to complement the reablement program skill. This would enable participants to use Alexa's integrated functions, for example, setting an ad‐hoc reminder to turn off a garden sprinkler, for cognitive compensation beyond the reablement program.

When I first set Alexa up, … I had to Google a fair bit and work out pros and cons … and how it all works.

(CP05)

Finally, the co‐design process revealed *adapting* personalisation of program content such as images, text and audio, were key to engagement and therapeutic efficacy. This was of particular importance given the need to target domains of cognitive impairments related to different stage, or type, of dementia.

…if somebody **tells** me a story, I'm only going to remember three per cent of it, if somebody **shows me a picture** then I'm going to remember 92 per cent of it.

(PLwD02)

### 3.2 Findings Across IDEAS Framework Stages

Key reablement theme findings generated through the IDEAS framework stages to inform the minimally viable reablement program delivered via DVA are summarised in Table 2. This iterative process demonstrated how initial concepts evolved through alignment with stakeholder feedback and technical feasibility assessment. Initial workshops provided an opportunity to understand the lived experiences and impact on daily function for PLwD.

I have to take medication, and if you get distracted, sometimes you just forget.

(PLWD01)

**Figure 2:**
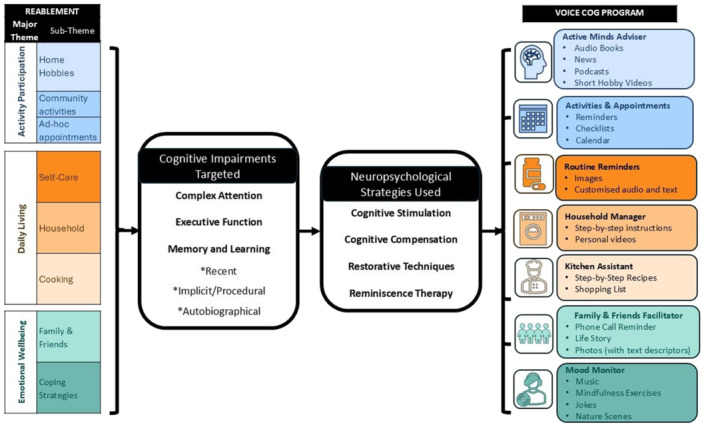
Schema illustrating reablement themes identified during co‐design, associated cognitive impairments targeted [58] and neuropsychological strategies used [57] in development of the personalised reablement program to be delivered via DVAs.

Involvement of key stakeholders helped identify cognitive impairments to target in relation to functional limitations and identify evidence‐based neuropsychological strategies [57] (steps 2 and 3 of the IDEAS framework) as illustrated in Figure 2. For example, challenges in performing home‐based hobbies were related to deficits in executive functioning skills as well as ‘procedural’ memory.

But someone's always got to start that activity a lot of the time…people can be quite apathetic and not have the ability to initiate …

(HP03)

Health Professional insights led to creative solutions incorporating other neuropsychological strategies, such as the ‘Kitchen Assistant’, a step‐by‐step recipe tool to support procedural memory impairments. Feedback emphasised the reminiscent therapeutic importance of using personal familiar recipes for individual flavour preferences, autobiographical recall and maintaining cultural identify.

…some of those simple recipes that they always cooked, like the biscuit or the apple pie. Those things that they always loved cooking in the past…The memory sort of comes back as they're going through that.

(HP03)

The iterative nature of the co‐design process considering divergent views allowed for innovative interventions that may not have been thought of otherwise. One example was the suggestion of replacing brain training games with learning jokes as a fun cognitive exercise.

…people think that doing brain games is somehow going to stop them from developing dementia or stop their cognitive impairment from getting worse, but I really feel strongly that that level of evidence is not there.

(HP06)

…. So that they get the joke, but in fact, then it's sort of a cognitive exercise in actually recalling the joke.

(HP02)

Notably, all co‐designed program goals were found to offer CP respite by relieving the cognitive load of ‘memory for two’, acting as ‘executive dysfunction secretary’ and providing respite by providing relaxing and cognitively stimulating entertainment in the home.

### 3.3 Prioritisation of Voice Cog Program Features and Functions

**Figure 3:**
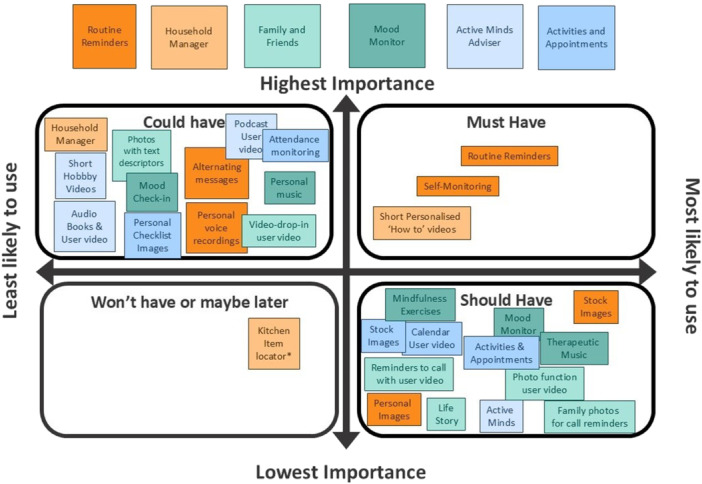
MoSCoW *prioritisation* of key program functions (Table 2) and technical features (Table 3): Consolidated interview feedback from people living with *early‐stage* dementia or MCI, and their CPs.

MoSCoW prioritisation conducted during semi‐structured interviews with people living with *early‐stage* dementia and their CPs, categorised program functions (Table 2) and technical features (Table 3) into ‘Must have, Could have, Should have or Won't have/maybe later’ (Figure 3). ‘Must have’ functions, those deemed essential and highly likely to be used, included the medication reminder function from the Routine Reminders goal. The importance of a self‐monitoring feature to compensate for short‐term memory loss was highlighted:

Sometimes I'll take it, and because I'm preoccupied with something else, I can't even remember the process of taking it.

(PLwD09)

Also prioritised as ‘Must have’ was the option of personalised ‘how to’ instructional videos for using household appliances, part of the Household Manager goal. This function was seen as critical in supporting cognitive impairments related to *learning and remembering how to use* new technology around the home, for example, TV remotes:

…what tends to happen is you manage the remote, then three weeks later, the next time I'm wanting to turn it on or whatever…and it'd be helpful to have someone else to tell me besides [spouse].

(PLwD06)

PLwD reported having difficulty with memory aids using traditional technology, when remembering ‘Activities and Appointments’ struggling with the cognitive demands of recalling procedural steps. Providing prompts to learn Alexa's calendar function that can be operated using simple voice commands was therefore viewed as a ‘Should have’ feature.

I'm finding this wall calendar works really well for me…. it's just been easier rather than…going to the computer and turning it on and going through all this stuff…. If I could **speak it,** that would be great.

(PLwD08)

Personal music and mindfulness functions in the ‘Mood Monitor’ goal were seen as ‘Should haves’, especially for those needing to cope with feelings of anxiety. This included the cognitive compensation of a reminder to take time for mood regulation:

…ABC Classics have a meditation on a Monday evening …. a friend who works as a music therapist told me about it. …. And there again, it's the reminder to do, it because I'd totally forgotten about it…

(PLwD08)

The ‘Reminder to Call’ feature of the ‘Family and Friends’ goal was also seen as a ‘Should have’ as a reminder of the benefits of reducing social isolation by keeping in touch:

… I think this isn't a good time to call now because they're at work…. And then I've forgotten, and another couple of days have gone past. Whereas I really think about them a lot and we want to talk to them.

(PLwD08)

The ‘Active Minds’ program goal was perceived as a ‘Could have’ ‘Lifestyle choice’ rather than reablement for people with early‐stage dementia, whose priority was maintaining ‘in‐person’ activities. While cognitive stimulation was valued in principle, there was concern expressed about potential hyperfocus on the YouTube videos function:

But what I'd be a bit scared of, just like the computer, is you go down a rabbit hole. And you're there all day with another distraction.

(PLwD08)

In addition to prioritisation of program's features, assistance in setting‐up the DVA was mentioned with support from a family member required. The suggestion of a technical ‘coach’ was proposed for future implementation.

…you take it out of the box, and you say ‘ooh…what do I do now?’, and that actually, for the person with, you know, some sort of cognitive challenges, is actually a big barrier.

(PLwD06)

### 3.4 Theoretical Framework Alignment

Application of the ICF framework (Table 4) demonstrated the co‐designed program covered a comprehensive range of cognitive reablement options, targeting daily activities, social relationships and emotional regulation. This mapping confirmed the program's alignment with established health frameworks, supporting its potential for addressing the multifaceted challenges of dementia.

**Table 4:** Reablement program goals mapped to the ICF categories.

Voice cog program	ICF codes and categories
Active minds: Audio books News Podcasts Short Videos	Domestic life (specific examples will depend on individual) D6505—taking care of plants outdoors D6500—making and repairing clothes
	Learning and applying knowledge d160 focusing attention d163 thinking d166 reading
Activities and Appointments	Community, social and civic life d910 community life d920 recreation and leisure General tasks and demands d230 carrying out daily routine
Family & friends Life Story Phone Calls Photos	Interpersonal interactions and relationships d750 informal social relationships d760 family relationships Communication d310 communicating with—receiving—spoken messages d330 Speaking d355 Conversation d360 using communication devices and techniques
Routine Reminders	Self‐care d550 eating d560 drinking d5702 knowledge about medication
Household manager	Domestic life d640 housekeeping d650 household tasks
Kitchen assistant Recipes Shopping Lists	Domestic life d630 preparation food d620 acquisition of goods
Mood monitor Jokes Mindfulness Music	Mental functions b152 emotional functions General tasks and demands d240 handling stress and psychological demands
DVA Use: Environmental factors, products and technology e115 products and technology for personal use in daily living e125 products and technology for communication	

## 4 Discussion

This study describes the involvement of key stakeholders, including PLwD and/or MCI, in the co‐design of a cognitive reablement program to be delivered via DVA. The progressive stages of the IDEAS framework led to a co‐designed cognitive reablement program supporting three functional domains: daily living, activity (and appointment) participation, and emotional wellbeing. In addition, PLwD or MCI, together with CPs and HPs, identified technical DVA feature requirements to optimise acceptability and usability, including (i) program activation, (ii) adherence and self‐monitoring, (iii) auditory and processing customisation, (iv) set‐up and training and (v) personalised multimodal content. Whilst the next step will report on pilot feasibility testing of the cognitive reablement program, a second co‐design phase carried out post‐feasibility testing included people living with *early‐stage* dementia to prioritise functions and features for a potential full‐scale trial. This prioritisation identified the self‐monitoring functionality of routine reminders such as medication, and personalised support for technical household appliances, as essential features for a potential full‐scale trial.

The first major reablement theme, to support daily living, particularly self‐care, emerged as a primary priority, aligning with the medication adherence reablement function of ‘understanding directions for medication use [d325]’ [59]. DVA features requested by PLwD to improve medication adherence included verbal alarm reminders with personalised prompts (e.g., number of tablets) and visual images (stock or personal). The critical need for such support is well‐established with research suggesting adherence can be as low as 17%–42% in PLwD [60]. Long‐term medication adherence significantly improved in PLwD or MCI using smart technology in a previous study, but results could have been influenced by weekly clinician visits [61]. Our study will require just one virtual clinical consultation to collaboratively agree goals before remotely broadcasting the co‐designed personalised cognitive reablement program via DVAs. Our approach is designed to minimise the requirement for health professional input, thereby increasing accessibility of support for PLwD or MCI to enable potential large‐scale implementation.

The second major theme to support activity participation aligns with current evidence demonstrating an association of physical and social activities with lower rates of cognitive decline [62, 63, 64]. Cognitive impairments in executive functioning and recent memory can affect the organisation required to attend these activities, a gap our intervention specifically targets [58]. Calendars as a cognitive compensatory tool can help maintain independence [65], but digital calendars using traditional technologies lack the customised multimodal reminders that DVAs can offer. Findings from digital calendars studies are limited by small sample sizes but learning technology for independent use can be a barrier for PLwD [66, 67]. Our co‐designed program provides a solution for regular scheduled activities with customisation of images and language, and voice‐control and interactive digital conversation minimising learning required. Although home‐based cognitively stimulating activities have not always been defined as reablement, their inclusion was grounded in the goal of enabling PLwD to continue engaging in meaningful leisure activities for enjoyment or relaxation. The Homeside study examined effectiveness of reading interventions, but only 15.9% of participants chose audiobooks, potentially due to participants living with more advanced stages of dementia [68]. Audiobooks have been found to reduce some behavioural and psychological symptoms of dementia [69]; however, few studies have examined outcomes for adults living with *early‐stage* dementia. Reminiscence therapy using internet‐based videos significantly improved cognitive function and apathy levels in people living with early‐stage dementia but required in‐person administration by a psychiatric nurse [70]. Our DVA program includes the opportunity for such reminiscence therapy to be administered remotely increasing scalability and reducing implementation costs.

The final reablement theme related to psychological well‐being and maintaining relationships with family and friends, reflecting a recognition of the psychosocial impact beyond cognitive symptoms. The emotional wellbeing module was included given the anxiety and depressive symptoms experienced across the dementia spectrum, which can negatively impact confidence and engagement in daily activities [71]. While a meta‐analysis found insufficient evidence on the effect of mindfulness‐based interventions for PLwD findings were limited by low‐quality studies [72]. An 8‐week mindfulness intervention involving adults with early‐stage dementia and their CPs reported improvements in relaxation and resilience justifying further research [73]. While the well‐being benefits of music for dementia have been well‐documented [74], people with dementia can lose the ability to initiate a device to play music [75]. A smart music player developed for PLwD successfully used spoken invitations to initiate music, but highlighted the need for personalised audio instructions [76]. Feedback from PLwD during our co‐design builds on those findings, by broadcasting customised instruction prompts for Alexa's integrated music function to play their personalised playlist. Lack of social engagement is a known risk factor for dementia [77], but fortunately current technology provides the capacity for remote video calls to maintain interaction. Phone adaption has successfully enabled PLwD to *answer* phone calls, but not the ability to remember when, and how, to make calls [78]. The co‐designed reablement program will address these cognitive limitations by providing personalised reminders and instruction prompts to make calls using Alexa's integrated phone and video function. Furthermore, the Family and Friends function will explore the use of a DVA to broadcast life stories, benefits of which include person‐centred therapy; autobiographical memory stimulation; cognitive stimulation in communication skills; increasing self‐esteem and preserving self‐identity and relationships with others [79]. The program will broadcast reminders to write Life Story chapters, with an opportunity to narrate their story depending on individual preferences and preserved abilities.

Adaptable technology functions to accommodate changing needs and abilities of people living with different types and stages of dementia was recommended by participants during co‐design. DVAs can overcome usability issues for motor‐based technology using keyboards, tablets and smartphones, by using voice for device control. Additionally, automated conversational announcements that explicitly state the desired behaviour (e.g., medication) overcome the digital notifications from traditional devices that are often overlooked. Lack of digital experience in older adults is associated with acceptability of technology, with training being a key facilitator to increase acceptability of novel technologies [39]. Our program will provide user videos along with personalised reablement support to increase digital competency, addressing a key barrier to adoption identified in previous research. The final personalised program aligns with ethical principles of dementia care of an individual, person‐centred home‐based program respecting the PLwD's autonomy to select two or three reablement goals from a holistic program enabling them to continue social engagement and meaningful activities [80]. Dignity is maintained by allowing the PLwD to inform personalised content (e.g., images), as well as frequency and timing of program broadcasts, tailored to their individual needs and capacities to achieve the intended benefit.

The application of the ICF framework to person‐centred dementia care can ensure community interventions provide meaningful reablement goals for PLwD [56, 81]. Our co‐design reablement program delivered by DVA achieves this comprehensive coverage by offering program goals across seven daily ICF activities. Furthermore, the program addresses the mental functions of emotional regulation and psychological wellbeing, as well as requiring cognitive skills for DVA use. The co‐designed program offers a holistic range of options for personalised cognitive reablement needs to support daily living, maintain leisure activities and manage emotional symptoms.

Prioritisation of functions and features by people living with ‘early‐stage’ dementia gave valuable guidance for stage‐appropriate reablement. The categorisation of Routine Reminders as an essential goal with self‐monitoring, suggests people living with *early‐stage* dementia place value on retaining autonomy rather than external surveillance. Likewise, this population felt Mood Monitor was relevant for provision of tools for emotional support, but continuous monitoring was not perceived as necessary at this stage. Activities and Appointments assistant and Family and Friends facilitator were perceived as important, which may reflect the need for executive functioning support for essential appointments and preference for maintaining social connection to family and community. The lower prioritisation of the Active Minds goal may indicate participants’ current reliance on existing cognitive abilities and established leisure routines. This exercise highlighted that people living with early‐stage dementia can meaningfully discriminate between essential, optional and unnecessary digital support features focusing on autonomy‐preserving reablement.

A primary strength of this study was the inclusion of PLwD throughout the co‐design process exemplifying a best‐practice approach. A recent review found only 14% of supportive technology studies actively included PLwD throughout the entire co‐design process [82]. The online and interactive methodology enabled participation from all over Australia and reduced participant burden associated with travel. Additionally, HPs were recruited from a diversity of backgrounds enhancing the representativeness of interventions incorporated. The depth of engagement required for co‐design requires significant time and resources, which resulted in a relatively small sample size and could limit generalisation of findings. However, participant feedback from future feasibility testing will also contribute to the program design prior to potential large‐scale implementation. Recruitment methods used in this study meant either the PLwD or CP had a reasonable level of digital literacy, so a future pilot trial should address this bias. CPs were included in this co‐design study as they are as a pre‐requisite for involvement in reablement programs. However, all CPs in this study were co‐habiting spouses, so feasibility testing should include those PLwD who have CPs that may live outside the home (e.g., adult children). Data on cultural and linguistic diversity were not collected, but workshop observations indicated limited diversity among PLwD/MCI and CPs. This should be addressed in future program evaluations to ensure acceptability to Australia's diverse population. Previous knowledge of DVA functionality and interventions, from researchers may have influenced findings; however, new functions and features were proposed by PLwD, CPs and HPs. Researcher's technical software knowledge may have shaped feasibility and must be acknowledged as a limitation to the co‐design process. For example, participant's ideas for password prompting for banking applications were not progressed due to security concerns illustrating the technical values tension from researchers.

To the best of our knowledge, this is the first co‐design of a cognitive reablement program delivered via DVAs involving PLwD, their CPs and HPs. The co‐designed reablement program will be tested in a pilot feasibility randomised controlled trial (ACTRN12623000898651) to assess acceptability and usability whilst exploring potential reablement effects. This will provide insights into DVA use for older adults to manage other health conditions, as well as the use of assistive technology for dementia.

## 5 Conclusions

This study illustrates the value of co‐design in engaging PLwD or MCI, their CPs, and multidisciplinary HPs, for developing a novel digital health intervention. The cognitive reablement program delivered via DVA included goals for maintaining independence in daily living activities, participation in leisure and community, managing wellbeing and interpersonal relationships. Technical functions and features were prioritised to ensure acceptability and usability of the DVA program. Application of the IDEAS framework enabled a better understanding of user needs to create a minimum viable product for further pilot feasibility testing and evaluation.

## Author Contributions

**Tracy Yiannis:** investigation, methodology, formal analysis, project administration, software, writing – original draft, writing – review and editing. **Helen Macpherson:** conceptualization, funding acquisition, writing – review and editing, supervision. **David Scott:** conceptualization, funding acquisition, writing – review and editing, supervision. **Michele Callisaya:** conceptualization, funding acquisition, writing – review and editing. **Caitlyn Gourlay:** investigation, methodology, writing – review and editing. **Ralph Maddison:** writing – review and editing. **Eugene Gvozdenko:** conceptualization, funding acquisition, resources, software, writing – review and editing. **Paul Jansons:** conceptualization, funding acquisition, investigation, methodology, formal analysis, writing – review and editing, supervision.

## Ethics Statement

This study was approved by the Deakin University Human Research Ethics Committee (DUHREC) Reference: 2022‐043.

## Consent

Online informed consent was obtained from all participants involved in the study.

## Conflicts of Interest

Eugene Gvozdenko is a director (unpaid position) of Great Australian Pty Ltd. Great Australian Pty Ltd provided in‐kind technical support for software and had a nonfinancial interest in the development of a voice‐based digital assistant software for telehealth. Great Australian had no input into this study's analysis, interpretation of the results or the decision to publish.

## Supporting information


Supporting File

## Data Availability

Relevant data are within the manuscript and supporting information files. De‐identified workshop and interview transcripts are stored by the research team but not publicly available due to privacy and ethical restrictions. The data that support the findings of this study are available from the corresponding author upon reasonable request.
