# Supplementary material for: Cognitive Reablement Using Digital Voice Assistants for People Living With Dementia or Mild Cognitive Impairment: A Co‐Design Study
Source: Health Expect. 2026 Aug 2;29(4):e70783. doi: 10.1111/hex.70783 (PMC13429101; doi:10.1111/hex.70783)
Supplement: Supplementary file 1 — Supporting File [file HEX-29-e70783-s001.docx]

**Supplementary Table 1** COREQ (COnsolidated criteria for REporting Qualitative research) Checklist

| **Topic** | **Item No.** | **Guide Questions/Description** | **Reported on Page No.** |
| --- | --- | --- | --- |
| **Domain 1: Research team and reflexivity** | | | |
| *Personal characteristics* | | | |
| Interviewer/facilitator | 1 | Which author/s conducted the interview or focus group? | 10 |
| Credentials | 2 | What were the researcher’s credentials? E.g. PhD, MD | 10 |
| Occupation | 3 | What was their occupation at the time of the study? | 10 |
| Gender | 4 | Was the researcher male or female? | 10 |
| Experience and training | 5 | What experience or training did the researcher have? | 10 |
| *Relationship with participants* | | | |
| Relationship established | 6 | Was a relationship established prior to study commencement? | 10 |
| Participant knowledge of the interviewer | 7 | What did the participants know about the researcher? e.g. personal goals, reasons for doing the research | 10 |
| Interviewer characteristics | 8 | What characteristics were reported about the inter viewer/facilitator? e.g. Bias, assumptions, reasons and interests in the research topic | 10 |
| **Domain 2: Study design** | | | |
| *Theoretical framework* |  |  |  |
| Methodological orientation and Theory | 9 | What methodological orientation was stated to underpin the study? e.g. grounded theory, discourse analysis, ethnography, phenomenology, content analysis | 6 |
| *Participant selection* |  |  |  |
| Sampling | 10 | How were participants selected? e.g. purposive, convenience, consecutive, snowball | 6-12 |
| Method of approach | 11 | How were participants approached? e.g. face-to-face, phone, mail, email | 6-12 |
| Sample size | 12 | How many participants were in the study? | 6-12 |
| Non-participation | 13 | How many people refused to participate or dropped out? Reasons? | 6-12 |
| *Setting* | | | |
| Setting of data collection | 14 | Where was the data collected? e.g. home, clinic, workplace | 6-12 |
| Presence of non-participants | 15 | Was anyone else present besides the participants and researchers? | 6-12 |
| Description of sample | 16 | What are the important characteristics of the sample? e.g. demographic data, date | 6-12 |
| *Data collection* |  |  |  |
| Interview guide | 17 | Were questions, prompts, guides provided by the authors? Was it pilot tested? | 6-12 |
| Repeat interviews | 18 | Were repeat interviews carried out? If yes, how many? | 6-12 |
| Audio/visual recording | 19 | Did the research use audio or visual recording to collect the data? | 6-12 |
| Field notes | 20 | Were field notes made during and/or after the interview or focus group? | 6-12 |
| Duration | 21 | What was the duration of the interviews or focus group? | 6-12 |
| Data saturation | 22 | Was data saturation discussed? | 6-12 |
| Transcripts returned | 23 | Were transcripts returned to participants for comment and/or | N/A |
| **Domain 3: analysis and findings** | | | |
| *Data analysis* |  |  |  |
| Number of data coders | 24 | How many data coders coded the data? | 16 |
| Description of coding tree | 25 | Did authors provide a description of the coding tree? | 16 |
| Derivation of themes | 26 | Were themes identified in advance or derived from the data? | 16 |
| Software | 27 | What software, if applicable, was used to manage the data? | 16 |
| Participant checking | 28 | Did participants provide feedback on the findings? | 16 |
| *Reporting* |  |  |  |
| Quotations presented | 29 | Were participant quotations presented to illustrate the themes / findings? Was each quotation identified? e.g. participant number | 16 |
| Data and findings consistent | 30 | Was there consistency between the data presented and the findings? | 17-22 |
| Clarity of major themes | 31 | Were major themes clearly presented in the findings? | 17-22 |
| Clarity of minor themes | 32 | Is there a description of diverse cases or discussion of minor themes? | 17-22 |

**Supplementary Table 2** Co-Design Workshops and Semi-Structured Interview Guides

| Workshop/Semi-Structured Interview | Key activities/components |
| --- | --- |
| Workshop 1 a & b (PLwD & CPs):   - Understanding lived experiences - Identifying cognitive impairments | - Introductions & Ice breaker activity: Experience of digital voice assistants - What is Cognitive Reablement? - Activity One: Sharing of successful cognitive reablement strategies - How could a Digital Voice Assistant be used in Reablement? - Activity Two: What cognitive reablement goals would make the most difference to you? - Activity Three: Cognitive Reablement plans – steps, challenges, support needed. |
| Workshop 2 (PLwD & CPs):   - Brainstorming Ideas | - Examples of how DVAs could support Cognitive Reablement Goals identified in Workshop I presented - Voting on usefulness of examples - De Bono’s Thinking Hats used to brainstorm ideas in relation to examples   - Benefits   - Associated Risks   - Creative ideas to overcome these risks |
| Workshop 3 a & b (PLwD & CPs)   - Prototypes feedback - Digital functionality & features | - Prototype Video Demo of Cognitive Reablement Goal Program via DVA from Workshop II - Discussion & ideas to improve - Feedback on technical usability of program e.g. initiation, format, training |
| Semi-Structured Interviews (HPs)   - Prototype Feedback | - Introduction to Cognitive Reablement via DVA Study - Video Demo of Cognitive Reablement Goal Program via DVA incorporating feedback from Workshop III - Discussion & feedback on usefulness   - Possible Barriers & ideas to overcome these   - Other features or functions to improve the program |
| Semi-Structured Interviews (Early-stage PLwD & CPs)   - User feedback: Interest & Usability | - Co-designed Reablement Goal Program Video Demonstrations - Explanation of MoSCOW (Must-have, Should-have, Could-have and Won’t-have/Not Yet’) prioritsation - Rating of co-designed Reablement Program Goals, Functions and Features to inform possible larger trial |

**Supplementary Table 3** Illustrative quotations supporting VOICE-COG Program modules

| Active Minds Adviser | Audiobooks | “PLwD03 reads; she’s always been a strong reader, but I know she can’t stay with it, you know, after a paragraph or so...” CP03  “I love the fact that you can have an audio book, and Alexa will know which chapter you need to read next or to listen to next.” CP05  “If PLwD03 was trained in it, it’d be fantastic…. PLwD03’s always enjoyed reading….and now she still reads but only reads the first page and then picks up another book...” CP03 |
| --- | --- | --- |
|  | News | “I think those are great interventions, the first three, the listening to the news, podcast, audio books.” HP06 |
|  | Podcasts | “And with any kind of cognitive intervention anyway, it’s going to be best if it’s sort of like imbedded within a system, within stuff that they already know. So, if it’s something like listening to the news or a podcast in a topic of interest, they are going to be drawing on things that they already know to interpret and understand that information.” HP06  “The concept is obviously great and for brain health keep them engaged in meaningful activity is huge.” HP05 |
|  | Short Hobby Videos | “I do have a little garden so that’s a big interest of mine, so I really enjoy looking after the garden, that’s something I do every day just even if it’s looking at the plants or picking a few flowers for the house.” PLwD01  “...whether there was anything in there in terms of sort of hands-on hobbies? Because that’s often one of those things that goes, so whether there was any YouTube clips of anything around that?” HP08  “I can imagine if someone has difficulty initiating these tasks or remembering to do them or maybe finding some of the resources that they might need, that being a really helpful thing.” HP05  “And maybe even like interesting documentaries, like David Attenborough documentaries, because those are all things that are stimulating, they are engaging and they require some level of cognitive engagement” HP06 |

| Activities and Appointments | Reminders | “So, you know, it’s kind of a more naturalistic form of practising memory, of keeping engaged with the community and what’s going on and being able to have, you know, conversations with people and being aware of what’s happening.” HP06  “We like to have at least one outing a day where we go out and do something, so it’s just good to connect….and the people around us, our community.” PLwD01  “Basically, exercise is our big thing, we walk as much as we can, we swim every day… PLwD03 with her noodle” CP03  “...and it just keeps her going on a Tuesday afternoon and a Thursday afternoon, to go to gym and do swimming.” CP05  “Do you have the Alexa help with any scheduling of that -reminders of calendars?” “I’m his Alexa.” CP04 |
| --- | --- | --- |
|  | Checklists | “We have a little checklist every day before we go out of the house, so I don’t forget the bus ticket or the mobile phone or the keys.” PLwD01  “Yeah, that’s great. Especially the reminder on what to take, that would be super helpful.” HP06 |
|  | Calendar | “So, any appointments that are not the regular things that you do every day…dental or doctor or chemist.” PLwD02  “Well for me just a verbal reminder of say dental appointment or doctor's appointment because they're the ones- even if you put them in your calendar, you can forget.” PLwD01  “I can see massive benefits for my husband with this.. although I put it into his calendar on his iPad sometimes, he doesn't look at it” CP04  “PLwD04’s calendar on his iPhone and his iPad is linked with mine, so everything I put into my calendar goes directly into his as well and that's where he gets his reminders. He also likes to write everything down on a paper calendar, so he's got a double prompt there. But I can see [to] somebody who doesn't have an iPhone or a smartphone or an iPad, this would be extremely useful.” CP04 |
| Routine Reminders | Images | “I really liked that one about medication because it was pointing to the drawer, I did like that for medication” PLwD02  “…if somebody *tells* me a story, I’m only going to remember three per cent of it, if somebody *shows* me a *picture* then I’m going to remember 92 per cent of it” PLwD02  “I have a blue tablet, and a brown tablet so blue tablet have to take morning and night and the brown tablet at night.” PLwD02  “I think a reminder for drinking water, fluids is really paramount. I have a lot of clients who end up, and one today, who's ended up in the emergency department because they're only drinking 500 mils of fluid maximum a day. So I think a water reminder, like putting a reminder on the tap is fine, but having a regular reminder to drink water would be amazing and I think really beneficial, especially early in their - with MCI or early dementia because hopefully it then becomes a pattern and it becomes a routine and that they actually continue with that as they go along their journey.” HP05  “I mean, I’ve got patients, I mean, who just don’t drink and so I’ve tried to set out a – the families will go and fill up a jug, a litre jug of water. The idea is that we just – they said when they make a telephone call say, “Have you had any more water and, you know, is the jug empty? Have you drunk your litre for the day?” They can drink whatever else that they like, but as long as they drink a litre of water we sort of know that they’ve probably had enough.” HP03 |
|  | Audio and Text | “I used to take four tablets in the morning and then one in the afternoon or in the evening, so I had to organise basically reminders for the four in the morning and make sure I was very specific and then 7.30 that night for just the one and the specific tablet itself. So, you've got to make sure that you actually detail the actual quantities and the actual specific tablet in the reminder.” PLwD02  “With myself it's important I take my medication every day. If I forget one day, then I could have a seizure, so I really find it valuable to have a reminder and I do have one on my phone at the moment which if I do forget then it prompts me. It tells me which is great because I haven't forgotten to take it and CP01 also reminds me at certain times. If I got caught up with something, you get distracted and then that's when you're likely to forget. So, the positive is that I won't forget if I have a reminder.” PLwD01  “If they say no, then you can be like don't forget muesli is in the cupboard, how about muesli and a nice yogurt, or how about an egg on toast, and those suggestions would be predetermined by them as well” HP02  “I think it's clear, concise, very understandable and the prompting is fantastic.” CP04 |
| Household Manager | Step-by-Step Instructions | “We’ve got a relatively new washing machine. It’s very sophisticated, and it’s got lots of programs on it. PLwD04 likes to do the washing, and hang it out, and bring it in, because I can no longer physically hang it out and bring it in, so he’s my carer in that way. But he sometimes forgets what program he should use for different types of washing that we separate into piles. So, having Alexa to help him do that, if he says, “I’ve got towels, what program do I use? I have sheets, which program do I use?” I would find that extremely helpful, because if I’m not here, he may put the wrong program in, and mess something up. Like put woollens in a hot wash, or something like that.” CP04 |
|  | Personal Videos | “Can I just say my biggest problem perhaps is using the remotes to record things on the television, at the moment CP01 does it all, but I’m terrified that if he’s not able to do it I wouldn’t be able to do it. So, it’s a matter of being able to record, you know, like on Foxtel or something like that?” PLwD01  “Well just the procedures or the steps that it takes to what I need to do because there’s two remotes and knowing which one you use first and which one you turn on first.” PLwD01 |

| Kitchen Assistant | Step by Step Recipes | “I can see the recipe demonstration and that application being really, really useful in Commonwealth home support packages for people with home care workers, because one of the activities that I ask them to do, for those that are particularly wanting to do recipes, is to come in, to take them shopping, to do their shopping to buy the ingredients, and then to come home and actually help them cook the meal, rather than just going straight to Meals on Wheels or Light ‘n’ Easy, which the most recent thing is supplying Light ‘n’ Easy microwavable meals. So, I can see a huge benefit from that sort of thing, not just from the individual’s perspective and their carer but also from the home care workers being able to help them with that particular task.” HP05 |
| --- | --- | --- |
|  | Shopping List | “PLwD04 is still working on a lot of levels really well. But one of the things he loves to do is food shopping. He will write himself a list, but his writing has got really bad since he’s been diagnosed with dementia. He’s got a very pronounced tremor. So, when he gets to the shop, he very often forgets what it is he’s supposed to be buying, because he can’t read it. So, now I’ve heard all this, and I know that we can do a shopping list with Alexa, where I can actually get it typed in, so he knows which cereal to buy and how much to buy, and that sort of thing, that’s going to be enormously helpful for us.” CP04  “...just a shopping list for me. I think that'd be - I tend to write it down and then I forget and all that sort of thing so if I had something as I just said [it] and it would be there, yes, would be great.” PLwD01 |

| Family & Friends Facilitator | Phone Call Reminder | “The reminders, the pictures because I think the pictures are obviously important you know names go pretty quickly. Faces are you know in terms of memory obviously are stick for longer.” HP08  “..the family and friends’ stuff, and that communication is really great, particularly if it is face based and by voice.” HP02  “So [name] can’t remember which one of her beautiful daughters she’s talking to half the time. But I think she certainly does if there’s a face.” HP02  “I think the reminders with the calls is - I can imagine that being quite helpful, especially if like initiation and recall is a challenge.” HP05  “I have a very wide and extended family, so the idea of the social engagement thing is we make a point to contact each member of the family on their birthday, so you keep a record of each of their birthdays, and that way you're still maintaining contact.” PLwD01 |
| --- | --- | --- |
|  | Life Story | “The other thing which I’m redoing again is a Book About Me, from when she was born, basically, to her early days, school and activities, and so forth. That was in A5, and I’ve converted that now into A4, making it a lot bigger, and adding on the extensions, the grandkids and so forth. So, that’s something that could also be put into the Alexa app, through the photos, and history, and memory of the person with dementia.” CP05  “When can I get started on it? It’s really good. I’ve got a similar sort of thing, you know, a book about me on PLwD05, you know, right from when she was born and everything else; the highlight all the way through, and trips overseas and all that, so perfect. Great.” CP05  “At the end of this program they actually collate everything into a book with photographs that you send... So very similar idea to this. But unfortunately, PLwD04 has lost his long-term memory as well as his short-term memory, because the idea was that we would write my story one week and PLwD04’s story the next, but he just can't remember, I remember more about his past than he does from things his mother has told me.” CP04  “I like it, it's amazing. Just from my own personal view, I love what I'm doing with story worth because I would never sit down and say I'm going to write my book because I'm busy, but this is just doing one chapter at a time and I'm prompted about what to write about, so it's really good.” CP04  “It's a great idea and I'm going to do it for myself now that you've prompted me, just with a photo of maybe every decade of my life… My mother is 97 and she's sort of losing her memory after a couple of strokes. But when she was very lucid, she actually wrote a book called Mon histoire which is French for my history. She has all her photos and everything that happened in her life. I've sort of forgotten a lot of the things that happened, so I go back to her book, so it reminds me of my childhood and her life. So, it's a wonderful idea.” PLwD01 |
|  | Photos | “Sometimes even a story that you’ve heard, it can be too much, so maybe having a couple of different options where one is more narrative, it’s got that nice, “This is your story” but maybe having another option for a day that’s not such a good day of, I don’t know... some photos.” HP01 |

| Mood Monitor | Music | “It looks good. I think music is powerful. And I have recently done some research on music therapy as well. So yeah, they're all good things.” HP01  “Well, we've used it for a fair bit of music, and my wife likes Janis Joplin so I usually say "Alexa, can you play Janis Joplin" and then I go out in the backyard or in the garden and she relaxes. She enjoys that type of music. So, it's been very relaxing for her, and she sits there and listens to it. So, it's just very soothing and a very good environment for her.” CP05  “I use music every day, we have music in the background, but music is so important even when we're walking or doing anything around the house.” PLwD01  “…we always have music in the background, so that’s something we love, and I love the way Alexa can pick a tune that you might love, and it’s a great way to start the day with the music.” CP04 |
| --- | --- | --- |
|  | Mindfulness Exercises | “I think I'd like to get PLwD04 more involved in doing something like this because he tends to sit down in front of the television. I think this would be something he could do during the day just for half an hour or so which would be good for his mental health.” CP04  “I think if my husband was home alone and I had preset this for him I think he wouldn't feel quite - sometimes he feels really lost if I'm not here and if there was something that could help keep him centred and calm, I think that would be very helpful.” CP04  “But I do like this box breathing bit here, I think that's really important because sometimes he gets anxious” CP04 |
|  | Jokes | “The other thing is we try to do the joke a day as well, just got to keep the humour there. I have an exchange with one of my sisters, she sends me a joke, I send her a joke. So that's wonderful to maintain the humour.” PLwD01  “In the joke function do you have the option of saying, “Hey, remind me about that joke, or remind me – can I – can I remember that joke because I’m going to tell it my grandkids when they”, you know, something like that. So that they get the joke, but in fact then it’s sort of a cognitive exercise in actually recalling the joke. If it’s not too long. I mean, it’s great. Can you imagine going to the men’s shed and just, there’s my joke for the day” HP02 |
|  | Nature Scenes | “we like to have at least one outing a day where we go out and do something, so it’s just good to connect with nature” PLwD01 |

PLwD: Person living with dementia

CP: Care Partner

HP: Health Professional
